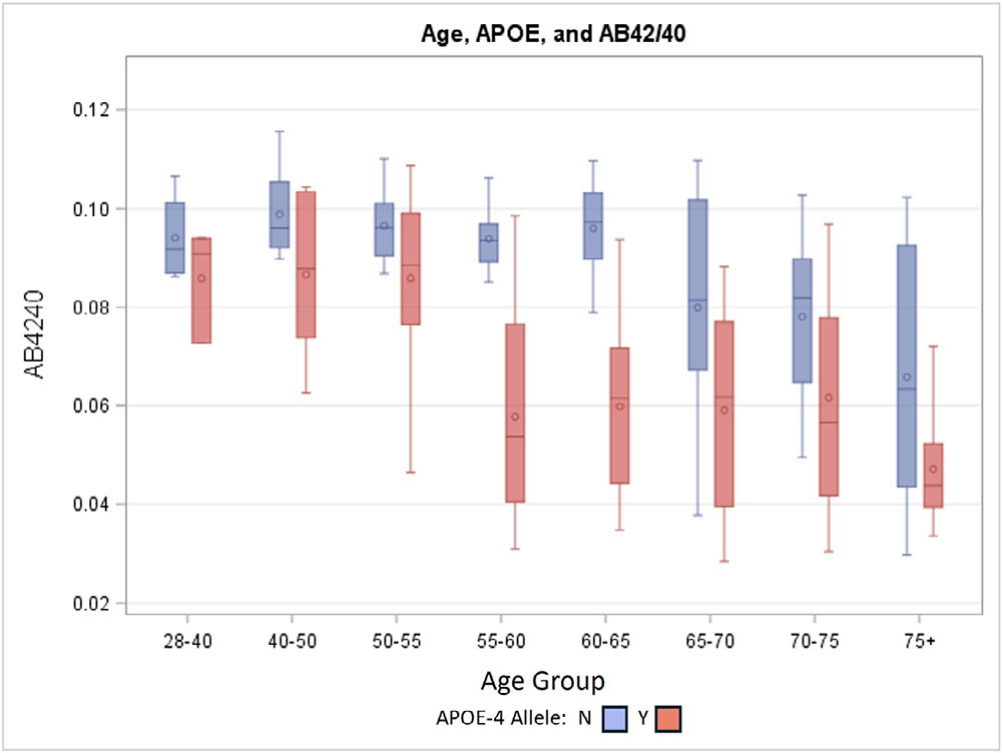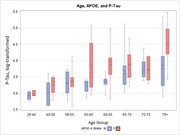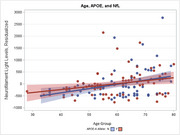# Demographic and Clinical Correlates of Biomarkers for Alzheimer's Disease and Related Dementias in the Duke‐UNC ADRC

**DOI:** 10.1002/alz70856_098089

**Published:** 2025-12-24

**Authors:** Patrick J Smith, Kim G Johnson, Heidi L Roth, Guy G Potter, Sara Patillo, Weili J Lin, Allen J Song, Miles Berger, Richard J O'Brien, Andy Liu, Michael W Lutz, Sheng Luo, Andrea Bozoki, Kathleen A. Welsh‐Bohmer, Gwenn A Garden, Heather Whitson

**Affiliations:** ^1^ University of North Carolina at Chapel Hill, Chapel Hill, NC, USA; ^2^ Neurology Department, Duke University Medical Center, Durham, NC, USA; ^3^ UNC, Chapel Hill, NC, USA; ^4^ Duke University– Joseph and Kathleen Bryan Alzheimer's Disease Research Center, Durham, NC, USA; ^5^ Duke University Medical Center, Durham, NC, USA; ^6^ Duke University, Durham, NC, USA; ^7^ Duke University School of Medicine, Durham, NC, USA; ^8^ Duke Department of Neurology, Durham, NC, USA; ^9^ University of North Carolina, Chapel Hill, NC, USA

## Abstract

**Background:**

Cerebrospinal fluid (CSF) biomarkers for Alzheimer's Disease and Related Dementias (ADRD) have gained widespread usage in clinical diagnosis and treatment, but their utility among middle‐aged individuals has yet to be fully elucidated. We examined ADRD biomarker profiles across the lifespan among adults in the Duke‐UNC ADRC cohort.

**Method:**

We examined demographic and clinical correlates of ADRD biomarker profiles among participants enrolled in the Duke‐UNC ADRC (*n* = 243) who underwent CSF biomarker assessments (*n* = 162). CSF markers included Aβ42/40, phosphorylated tau (*p*‐tau181), and neurofilament light (NfL). We also characterized biomarker positive individuals using a cutoff of AΒ42/40 ≤ 0.062. Individuals with at least one APOE risk allele were considered to have elevated APOE risk. Linear regression models were used to characterize correlates of ADRD biomarkers, controlling for age, education, gender, race, and APOE genotype.

**Result:**

Participants ranged from 28 to 80 years of age (mean = 59.8 years [SD = 11.4]), were mostly female (*n* = 111, 69%) and white (*n* = 129, 20%), nearly half had ≥ one APOE risk allele (48%), and 36 (27%) were biomarker positive. Age and APOE status were the most robust correlates of biomarker levels. Older age associated with lower AΒ42/40 (*B* = ‐0.44, *p* <.001), and greater *p*‐tau181 (*B* = 0.53, *p* <.001), and NfL (*B* = 0.30, *p* <.001). APOE status similarly associated with lower AΒ42/40 (*B* = ‐0.45, *p* <.001), and greater *p*‐tau181 (*B* = 0.24, *p* = .001). Visual inspection of ADRD biomarker levels suggested that AΒ42/40 levels approached or exceeded clinical thresholds in some individuals beginning in midlife.

**Conclusion:**

Age and APOE risk robustly associate with ADRD biomarkers and this association may begin in middle‐age.